# *Grammatophyllum speciosum* Ethanolic Extract Promotes Wound Healing in Human Primary Fibroblast Cells

**DOI:** 10.1155/2018/7836869

**Published:** 2018-10-21

**Authors:** Saraporn Harikarnpakdee, Verisa Chowjarean

**Affiliations:** ^1^Cosmeceutical Research, Development and Testing Center, College of Pharmacy, Rangsit University, Pathum Thani 12000, Thailand; ^2^Department of Industrial Pharmacy, College of Pharmacy, Rangsit University, Pathum Thani 12000, Thailand; ^3^Department of Pharmaceutical Technology, College of Pharmacy, Rangsit University, Pathum Thani 12000, Thailand

## Abstract

*Grammatophyllum speciosum* is a plant in Orchidaceae family which contains a variety of phytochemical compounds that might be beneficial for medicinal use. This study aimed to evaluate the activity of pseudobulb of* G. speciosum *extract (GSE) in wound healing processes in human primary fibroblast cells along with* in vitro* antioxidant activity and total phenolic content of GSE. Scratch wound healing assay indicated that GSE was capable of increasing migration rate after 6 and 9 hours of treatment. Besides, the extract was able to scavenge DPPH, ABTS, and superoxide anion radicals indicating the antioxidative property of GSE. This study suggested a novel role of the of pseudobulb extract of* G. speciosum* as a wound healing enhancer. The results from this study might be beneficial for the development of further novel active compounds for skin wound healing.

## 1. Introduction 

Wound healing process is divided into four phases classified as vasoconstriction and coagulation, acute inflammation, cellular proliferation, and wound remodeling [1]. Briefly, coagulation causes the development of platelet thrombosis and fibrin clot leading to the recruitment of neutrophils and macrophages which leads to inflammatory response. Growth factors and proinflammatory cytokines are then released to activate cells which involve antimicrobial process such as keratinocyte, endothelial, and fibroblast. During inflammatory response, reactive oxygen species (ROS) are produced to defend cells against bacteria and microorganism invasion. Histamine and other factors leading to an increase in vasodilatation are also released. Cellular proliferation phase is started by the accumulation of extracellular matrix (ECM), including collagen and fibronectin which are produced from fibroblasts. The proliferation of endothelial cells stimulated by vascular-promoting growth factors leads to an increasing amount of blood vessels. Finally, reepithelializing mechanism and tissue remodeling are then started. At this process, fibroblasts are activated by keratinocytes to synthesize growth factors to regulate their proliferation. A noncellular scar, and cross-linked collagen matrix will substitute fibroblast-rich granulation tissue and provide scar tensile strength or intact skin. During the tissue remodeling phase, a decrease in cellularity from the apoptosis of myofibroblasts, endothelial cells, and inflammatory cells will lead to the process of impaired wound healing. This impairment may be caused by uncontrolled inflammatory, infection, and overproduction of ROS. Excessive ROS production could damage ECM protein and cellular function of fibroblasts and keratinocytes, resulting in slow wound healing process [1–4]. The plant extracts with potential for skin protection from its antioxidant property might be useful for the acceleration of tissue wound healing [5].

*Grammatophyllum speciosum* Blume is a plant in Orchidaceae family mostly found in Southeast Asia. The pseudobulb extract of* G. speciosum* was used for relieving pains from scorpion venom (*Heterometrus laoticus*). In addition,* G. speciosum* ethanolic extract (GSE) was reported to have potential for an increase in stem cell phenotypes of human keratinocytes [6]. Moreover, the extract also had an ability to protect the cells against superoxide anion-induced cell death [6].* G. speciosum* contains various phytochemical compounds such as glucosyloxybenzyl derivatives, grammatophyllosides, cronupapine, vandateroside II, gastodin, vanilloloside, orcinol glucoside, and isovitexin [7]. Due to their benefits, these phytochemicals in this plant may be used as medicine; however, the effect of* G. speciosum* on tissue wound healing has never been investigated.

This study examined the potential wound healing effects of GSE in human primary skin fibroblast cells. Also, the in vitro antioxidant activity of GSE was evaluated for its ability to scavenge DPPH, ABTS, and superoxide anion radicals. The results from this study might be beneficial for the development of further novel active compounds for tissue wound healing treatment.

## 2. Materials and Methods

### 2.1. Plant Material Collection and Extraction

Fresh pseudobulbs of* G. speciosum* Blume were collected from the area of Khao Hin Sorn Royal Development Study Center, Chachoengsao Province, Thailand. Dry pseudobulbs of* G. speciosum* were ground up and macerated for 3 days in ethanol (1:9 w/v) at 25°C for a total of 3 times. The extract was then filtered and evaporated under vacuum pressure at a temperature below 40°C.

### 2.2. Quality Control of* G. speciosum* Bulb Extract

For reproducibility data of the extract, determination of gastrodin content which is a major compound of* G. speciosum *extract from three different batches was performed using a rapid high-performance liquid chromatography method under the conditions using gradient elution with a mobile phase of acetonitrile–water, as previous described by our group [8].

### 2.3. Total Phenolic Content Determination

A total phenolic content was determined using Folin-Ciocalteu assay [9]. In a 96-well plate, the reagent was mixed with the samples to which Na_2_CO_3_ was consequently added. The mixture was incubated for 1 hour and the absorbance was measured using the UV-spectrophotometer at 765 nm. The total phenolic content was compared with the epigallocatechin gallate (EGCG) and expressed as g of EGCG equivalents (gEGCG) per 100 g of GSE extract. The experiment was repeated in triplicate.

### 2.4. Assessment of Antioxidant Activity

#### 2.4.1. DPPH Assay

The 2,2-diphenyl-2-picrylhydrazyl (DPPH) radical scavenging activity of GSE was determined by using DPPH radical. The samples were added to 0.15 mM DPPH solution in ethanol in a 96-well plate. The mixture was incubated for 30 minutes in the dark at room temperature. The absorbance was measured using the UV-spectrophotometer at 517 nm. The results were expressed as % inhibition of DPPH. DPPH solution with the vehicle was used as a negative control whereas the vehicle without DPPH solution was used as a blank for background subtraction.

#### 2.4.2. ABTS Radical Scavenging Activity

Twenty micrometers of the samples were added to a 96-well plate before adding ABTS^•+^ solution 180 *μ*l. The solutions were mixed using a shaker and incubated in the dark at room temperature for 5 minutes. The absorbance of the ABTS•^+^ was measured using the UV-spectrophotometer at 750 nm. The results were expressed as %inhibition of ABTS•^+^ by GSE compared to the ABTS•^+^ solution with the vehicle used as the negative control. A 50% reduction of the ABTS•^+^ was calculated and presented in an IC50 value.

#### 2.4.3. Superoxide Anion Radical Scavenging (SOSA) Determination

Twenty micrometers of the samples was added to a 96-well plate before adding 20 *μ*l phosphate buffer, 80 *μ*l NADH, 80 *μ*l NBT, and 20 *μ*l PMS solutions. The solutions were incubated in the dark at room temperature for 15 minutes. The absorbance of the mixture was measured using the UV-spectrophotometer at 560 nm. The results were expressed as % inhibition of SOSA by GSE compared to the ABTS•^+^ solution with the vehicle used as the negative control. A 50% reduction of the superoxide anion radical was calculated and presented in an IC50 value.

### 2.5. Cell Viability Assay

Human primary skin fibroblast cells (ATCC® CRL 2097, USA) were cultivated in a 96-well plate for approximately 5 × 10^3^ cells/well density using 10% FBS-supplemented DMEM medium and were incubated in 37°C with 5% humidity and CO_2_ condition overnight. After the incubation, DMEM was replaced by 10% FBS DMEM containing 5–100 *μ*g/mL of GSE, except in control. The cells were incubated in 37°C, with humidified 5% and CO_2_ atmosphere for 24 h, respectively. Afterwards, 100 *μ*L of 3-(4,5-dimethylthiazol-2-yl)-2,5-diphenyltetrazolium bromide (MTT) solution (5 mg/mL in PBS) was used to replace culture medium in each well, and the plate was then incubated at 37°C, dark, in 5% humidity and CO_2_ condition for 3 h. After removing MTT solution, 100 *μ*L of DMSO was instead added to each well and a microplate reader was then used to read the absorbance at a wavelength of 540 nm. Cell viability was calculated into percentage and compared to the control sample.

### 2.6. Scratch Wound Healing Assay

The stimulatory effect of GSE on migration of human primary fibroblast cells was determined by the scratch wound healing assay. The cells were seeded at a density of 3 × 10^4^ cells/well in a DMEM culture medium supplemented with 10% FBS in 96-well plates and incubated for overnight. After the incubation, DMEM was completely removed and the adherent cell layer was scratched with a sterile yellow pipette tip. Cellular debris was removed by PBS rinsing. The complete medium with GSE or without GSE was then added, and the cells were incubated for 9 h. At 0, 6, and 9 h, the image of scratch area was recorded under bright field microscopy (10×). The wound area was measured using the Olympus DP controller software. The results were expressed as relative cell migrations by dividing the percentage change in the space of the GSE-treated cells at 6 and 9 h compared to 0 h in each experiment.

### 2.7. Statistical Analysis

Data from independent experiments were presented as mean ± SD. The statistical differences among the multiple groups were analyzed using the analysis of variance ANOVA), and the individual comparisons were performed by Scheffe's post hoc test. Statistical significance was accepted at* p* < 0.05.

## 3. Results 

### 3.1. Percent Yield and Appearance of* G. speciosum* Extract

**Figure 1:**
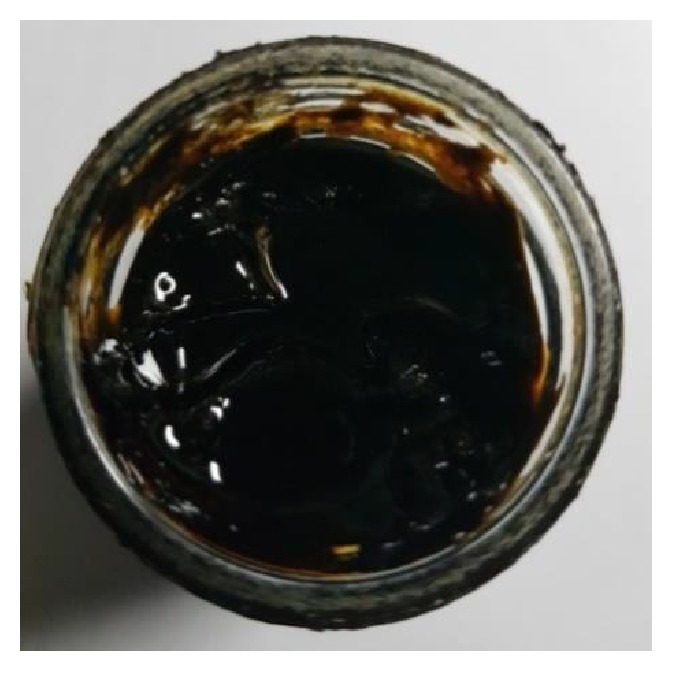
The ethanolic extract from* G. speciosum* pseudobulbs.

The crude extract of* G. speciosum* Blume pseudobulbs was 194.6 g accounted as 6.49% w/w yield compared to the initial dry weight. Crude herb of* G. speciosum* were ground and extracted according to the extraction process to obtain herb-to-extract ratio (HER) of 1:9. The* G. speciosum* extract obtained was highly viscous with a dark-brown color. The appearance of* G. speciosum* extract is shown in Figure 1.

### 3.2. Determination of Gastrodin Content by HPLC

**Figure 2:**
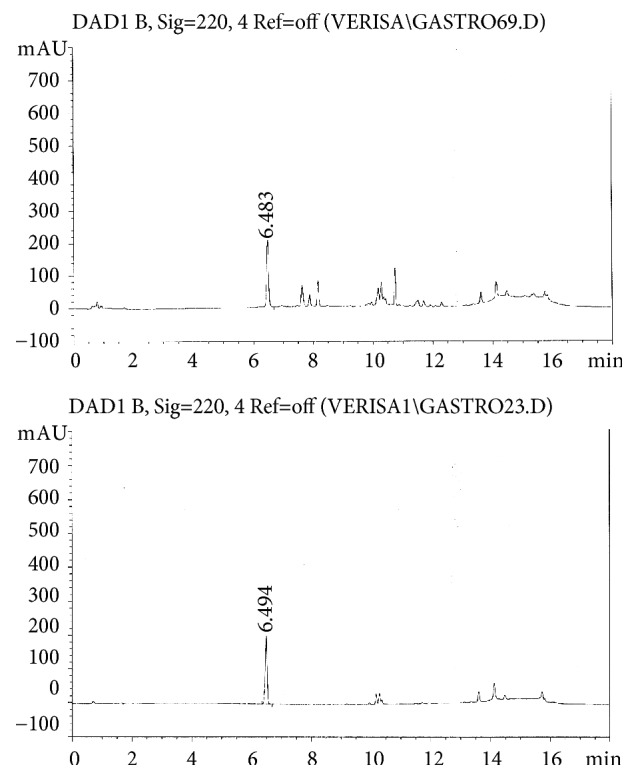
Typical HPLC chromatogram of the* G. speciosum* extract (top) and gastrodin standard (bottom).

The determination of gastrodin content which is a major compound of* G. speciosum* extract was performed using a rapid high-performance liquid chromatography method under the conditions using gradient elution with a mobile phase of acetonitrile–water. The retention time of* G. speciosum* extract samples and standard solutions were found to elute at 6.483 and 6.494 min, respectively (Figure 2).

The amount of gastrodin in 3 different batches of GSE was analyzed. The results showed that the amount of gastrodin from batch 1, batch 2, and batch 3 contained 63.62±0.2, 54.96±0.2, and 56.65±0.3 mg/g, respectively (Table 1), which was considered to be not significantly different from one another (*p *> 0.05). The result thus suggested that gastrodin levels were not significantly different among batches, indicating uniformity among batches collected and extracted from different harvest periods.

### 3.3. Total Phenolic Contents and Antioxidant Activity of the GSE

Phenolic compounds may directly play a role in the antioxidant effect. The GSE was determined for the total phenolic content using Folin-Ciocalteu assay compared to the standard compound EGCG. The total phenolic content of GSE was 48.2±0.4 mg EGCG equivalent/g.

The present study demonstrated the antioxidant capacities of GSE against the DPPH radical, in which DPPH is a free radical compound normally used for screening the radical scavenging effect.  Table 2 shows the percentage of the scavenging activity of GSE ranging from 24.8 to 55.7% with IC50 at 0.1 mg/mL. The percentage of the scavenging activity of ascorbic acid, the standard compound, was 21.7 to 76.3% with IC50 at 2.3 mg/mL. The IC50 of GSE was lower than that of ascorbic acid indicating that GSE has high antioxidant capacity than ascorbic acid.

**Figure 3:**
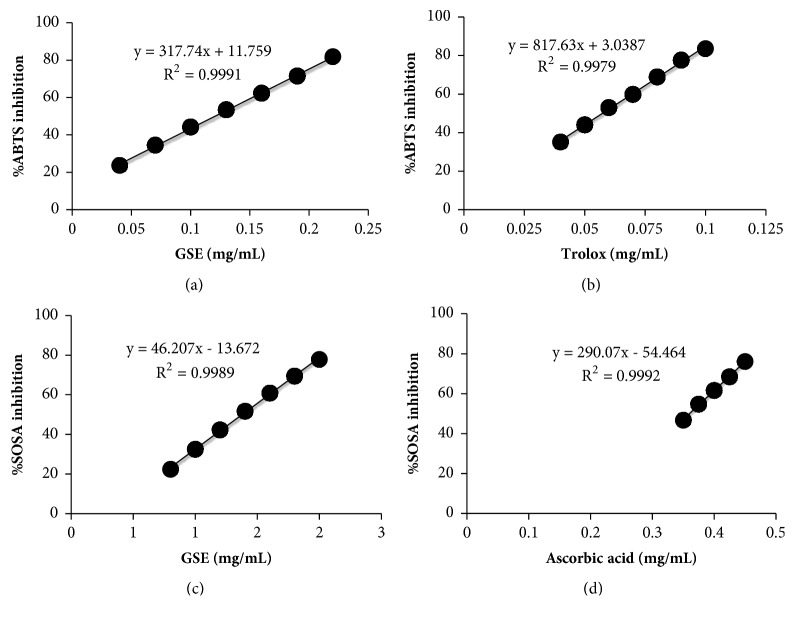
Total antioxidant activity. Total antioxidant activity of GSE and trolox. Effects of (a) GSE and (b) reference compound trolox on the decolorization of ABTS radical cation. Effects of (c) GSE and (d) ascorbic acid on SOSA inhibition were investigated. The percentage inhibition was plotted against the concentrations of the samples. Data are expressed as mean ± SD (n = 3).

The calculation of the total antioxidant activity was performed based on the decolorization of ABTS•^+^. Trolox was used as a standard compound. The results were expressed as %inhibition of ABTS•^+^ radical. GSE (Figure 3(a)) or trolox (Figure 3(b)) suppressed the absorbance of the ABTS•^+^ radical in a dose dependent manner. The IC50 of %inhibition of ABTS•^+^ of GSE and trolox were 0.12 ± 0.01 and 0.06 ± 0.01 mg/mL, respectively. Moreover, a further investigation was made to discover the antioxidant capacities of GSE against superoxide anion by determining SOSA. The IC50 of SOSA inhibition of GSE was 1.37 mg/mL (Figure 3(c)). However, the IC50 of GSE was higher than ascorbic acid (IC50 of ascorbic acid was 0.36 mg/mL) as presented in Figure 3(d).

### 3.4. Effects of GSE on Human Primary Fibroblasts Cells Viability

**Figure 4:**
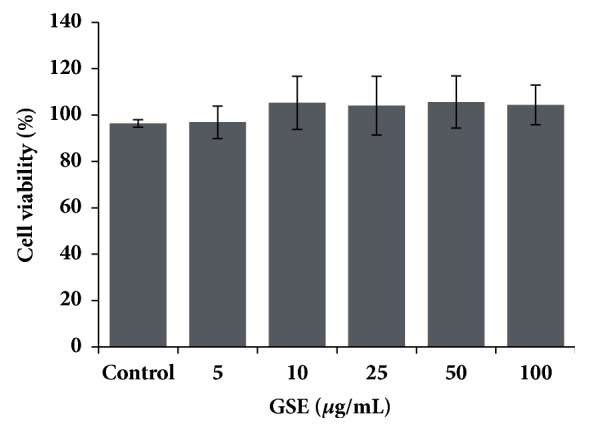
Effects of GSE on human primary fibroblast viability. Human primary fibroblast cells were treated with GSE (0–100 *μ*g/mL) for 24 h and tested for their viability using a MTT assay. The cell viability (%) was calculated to those of the untreated controls. Data are presented in mean ± SD (n = 3). *∗ p* < 0.05 versus untreated control.

An effect of GSE on cell viability was evaluated in human primary fibroblasts cells in different concentrations after 24 h, using MTT assay. The concentration of GSE 5–100 *μ*g/mL was shown to be noncytotoxic in primary fibroblast cells, and the cell viability was higher than 80% (Figure 4). Nontoxic concentrations were selected to study scratch wound healing assay.

### 3.5. GSE in the Enhancement of Wound Healing in Human Primary Fibroblasts Cells

**Figure 5:**
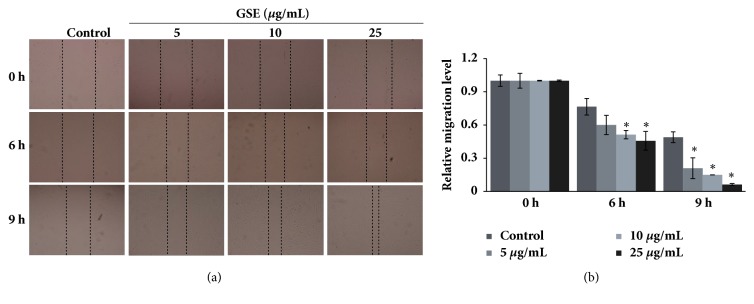
Effects of GSE on human primary fibroblast cell migration. Human primary fibroblast cells were treated with GSE (0–25 *μ*g/mL) for 9 h and subsequently tested for their migratory abilities by scratch wound healing assay. (a) Phase-contrast images (10×) captured at 0, 6, and 9 h and (b) the relative cell migration compared to that of the untreated controls. Data are presented in mean ± SD (n = 3). *∗ p* < 0.05 versus untreated control.

Human primary fibroblasts cell line was tested through the wound healing assay to determine the capacity of these cells to migrate after GSE treatment. Cell migration was determined at 6 and 9 h after exposure to GSE. GSE at a concentration of 5–25 *μ*g/mL increased the cell migration rates at 6 and 9 h when compared to 0 h (Figure 5(a)). The relative migration levels of human primary fibroblasts cells were presented in Figure 5(b), supporting that GSE significantly enhanced the migration rates after 6 and 9 h of GSE treatment.

## 4. Discussion 

Natural plant extracts can be beneficial for wound healing if their phytochemical has the antioxidant activities and free radical scavengers, as reported by many previous studies [5].* Caesalpinia mimosoides* extract contains phenolic contents and has a remarkable antioxidant activity leading to the enhancement of the wound healing property [10]. In line with that, this phenomenon was studied by treating GSE on human primary fibroblast cells to analyze its potential on wound healing.

Cell migration is an important process in the tissue formation phase of wound healing [1–3]. The wound healing assay is the method imitating a wound in vitro and investigating a cell migration rate. After a cell monolayer is destroyed by scratching, a loss of cell-cell interaction occurs, resulting in the initiation of cell migration and proliferation. In this study, GSE is able to increase a cell migration rate, indicating the wound healing enhancement.

The formation of reactive oxygen species (ROS), including hydrogen peroxide (H_2_O_2_), superoxide anion (·O2−), hydroxyl radical (·OH), lipid peroxides (LOOH), and their radicals (LOO·) occurred and accumulated in skin cells that expose to UVA and UVB (290–320 nm and 320–400 nm respectively). These ROS could break down cell membranes and induce skin inflammation, aging, and phototoxicity. Excessive amounts of these ROS might impair the skin wound healing process. In this sense, the plant extracts with antioxidant activity might be beneficial for the enhancement of the wound healing process [11, 12]. This study found that GSE has antioxidant activity, as determined by its ability to scavenge DPPH, ABTS and superoxide anion radicals.

Gastrodin, a phenolic glycoside [p-hydroxymethylphenyl-*β*-D-glucopyranoside], is found as a major active component in* G. Speciosum*. This study selected gastrodin as an active marker to control the quality of GSE due to its presence in significant amount, thereby facilitating detection and analysis. It was also used as an analytical marker for quantitative evaluation of* Rhizoma Gastrodiae* (Tianma) extracts using HPLC [13, 14]. Gastrodin is a potent antioxidant, which showed protective effects against osteoporosis linking to a reduction in ROS [15]. The antioxidant effect of GSE might be exerted from the ability of gastrodin in scavenging of ROS.

Bhat et al. and Pitz et al. found the enhancement of wound healing effects of* Caesalpinia mimosoides* extract and Jaboticaba fruit peel extract, respectively, probably caused by their phenolic contents [10, 16]. Likewise, the wound healing property of GSE might be from its phenolic compound.

## 5. Conclusion 

GSE has a potential to enhance the wound healing process of human primary fibroblast cells. Besides, in relation to the increase in wound healing property, the extract is able to scavenge DPPH, ABTS, and superoxide anion radicals. Hence, this study provides interesting effects of GSE for the first time on tissue wound healing enhancement that warrants further investigations and may provide useful information for further therapeutic applications.

## Figures and Tables

**Table 1:** Gastrodin contents of GSE at different batches.

Batch	No. 1	No. 2	No. 3
Gastrodin (mg/g)	63.62±0.2	54.96±0.2	56.65±0.3

**Table 2:** Antioxidant activity of GSE and ascorbic acid determined by the DPPH assay.

GSE		Ascorbic acid	
mg/mL	%Inhibition of DPPH radical	mg/mL	%Inhibition of DPPH radical
0.030	24.8 ± 0.5	1.0	21.70 ± 2.3
0.045	32.2 ± 2.2	1.5	32.50 ± 5.6
0.060	38.8 ± 2.1	2.0	45.52 ± 5.6
0.075	45.2 ± 1.2	2.5	56.52 ± 5.1
0.090	50.6 ± 2.0	3.0	67.59 ± 5.3
0.105	55.7 ± 1.1	3.5	76.28 ± 3.9

Values are presented in mean ± SD (n = 3).

## Data Availability

The data used to support the findings of this study are included within the article.
